# Classification-based repair techniques to correct tricuspid valve incompetence in Ebstein’s anomaly and long-term functional outcome

**DOI:** 10.1186/1749-8090-8-S1-O137

**Published:** 2013-09-11

**Authors:** R Hetzer, E Delmo Walter

**Affiliations:** 1 Cardiothoracic Surgery, Deutsches Herzzentrum Berlin, Berlin, Germany

## Background

We describe a repertoire of repair techniques according to types of Ebstein’s anomaly to correct tricuspid valve (TV) incompetence, and report the long-term and functional outcome.

## Methods

Sixty-eight (68) patients (mean age 26.9±7.3 years) with Ebstein’s anomaly (Types A=18, A=3, B=21, B-C=2, C=15, D=9) underwent correction of TV incompetence. The atrialized ventricle, the TV and subvalvar apparatus were inspected to analyze the precise morphology, and determine which leaflet was the most mobile. In all, the atrialized right ventricle (RV) is incorporated into the contractile RV by partial closure of the natural annulus using the most mobile leaflet for valve competence. Posterior annulorrhaphy was performed for Types A,B,C. The double orifice valve technique was employed mostly in types C and D.Sebening stitch, was applied in combination in most types. A combination of anterior and posterior annulorrhaphy was also performed for types A and B. In 3 patients with type C, additional bidirectional Glenn anastomosis was performed.

## Results

Mean follow-up duration was 13.25±1.3 (range 1-24.) years. Mean NYHA class improved from 3.4 to 1.3 (p<0.001). Severity of TV incompetence was reduced from 3.2 to 1.3 (p<0.001). Exercise tests demonstrated improvement in maximal oxygen uptake (p<0.02). Mean basal, middle and apical ventricular strain significantly improved to 25.7% (p<0.011), 23.7% (p<0.001) and 19.36% (p<0.05). Freedom from reoperation was 100% at 1 year and 92.9% at 5 and 20 years, respectively. Early and late mortality was 5.8% and 2.9% respectively. Overall survival rate was 94.2% and 91.26% at 30 days and 20 years, respectively.

## Conclusion

The various repair techniques, which preserve the atrialized chamber, and employed individually according to morphology, provide satisfactory long-term ventricular function and functional outcome even in severe types of Ebstein’s anomaly.

